# [Retracted] Isoquercitrin inhibits the progression of liver cancer *in vivo* and *in vitro* via the MAPK signalling pathway

**DOI:** 10.3892/or.2023.8510

**Published:** 2023-02-28

**Authors:** Guihong Huang, Bo Tang, Kun Tang, Xiaomin Dong, Jungang Deng, Luqin Liao, Zengzhen Liao, Hua Yang, Songqing He

Oncol Rep 31: 2377–2384, 2014; DOI: 10.3892/or.2014.3099

Subsequently to the publication of the above article, a concerned reader drew to our attention that the data panel shown in Fig. 7A for the 400 μM isoquercitrin experiment had previously appeared in Fig. 4A in another article published in the journal *International Journal of Oncology* [Tang B, Li Y, Yuan S, Tomlinson S and He S: Upregulation of the δ opioid receptor in liver cancer promotes liver cancer progression both *in vitro* and *in vivo.* Int J Oncol 43: 1281–1290, 2013], indicating that results that were purported to have been obtained under different experimental conditions had been derived from the same original source. Furthermore, concerns were also raised regarding the originality of some of the other data belonging to this figure.

Given the errors that were identified in the compilation of Fig. 7 in this article, the Editor of *Oncology Reports* has decided that this article should be retracted from the publication owing to a lack of overall confidence in the presented data. The authors were asked for an explanation to account for these concerns, but the Editorial Office did not receive a reply. The Editor apologizes to the readership for any inconvenience that might result from the retraction of this article.

